# Effect of training on knowledge, behaviors, and low back pain among marble workers: non-randomized experimental study

**DOI:** 10.3389/fpubh.2025.1725015

**Published:** 2026-01-23

**Authors:** Gülcihan Aybike Dilek Kart, Ayse Meydanlioglu

**Affiliations:** 1 Department of First Aid and Emergency, Vocational School of Health Services, Burdur Mehmet Akif Ersoy University, Burdur, Türkiye; 2 Health Sciences Institute, Akdeniz University, Antalya, Türkiye; 3 Department of Public Health Nursing, Faculty of Nursing, Akdeniz University, Antalya, Türkiye

**Keywords:** interactive education, low back pain, marble worker, printed material, public health nurse

## Abstract

**Background:**

Workers in physically demanding industries, such as the marble sector, are highly susceptible to low back pain. This study was conducted to determine the effect of the training given to the workers in a marble factory using different methods on knowledge and behavior for protecting low back health and low back pain level of the workers.

**Methods:**

This non-randomized experimental study with a pre-test, post-test, follow-up, and control group design was conducted with 135 workers. The data were collected with the Low Back Health Protection Information Form, Manual Handling Observation Form, Visual Analog Scale (VAS). *P* < 0.05 was considered statistically significant in the study.

**Results:**

Knowledge scores increased significantly in both intervention groups, with the highest effect observed in the interactive training + printed materials group (*p* < 0.001, η^2^ = 0.66). Manual handling behavior scores improved more than doubled in this group (*p* < 0.001, η^2^ = 0.92). Low back pain (VAS) scores decreased significantly in both intervention groups, with a larger reduction in the interactive training group (*p* < 0.001, η^2^ = 0.60).

**Conclusion:**

Interactive education is more effective in protecting the back health of workers, increasing the level of knowledge and behavior, and reducing low back pain compared to the use of printed materials alone. However, the use of printed materials also had a significant effect. Proper manual handling training for workers who lift heavy loads can reduce low back pain and increase knowledge and behaviors related to low back health.

## 1 Introduction

Low back pain (LBP) is one of the most common occupational musculoskeletal disorders worldwide and remains a leading cause of disability, reduced productivity, and economic burden (1). Workers in occupations requiring manual material handling—such as construction, mining, transportation, and manufacturing—experience particularly high rates of LBP due to repetitive lifting, bending, twisting, and physical overexertion (2, 3). Global disability years due to lower back pain related to occupational ergonomic factors increased by 40.6% from 1990 to 2021 (4). In Türkiye, prevalence is ~32% among adults (5), increases to 65% among industrial workers (6), and reaches up to 80% among mine workers (7). The marble industry, classified as a “very hazardous” sector due to its intense physical workload (8), presents particularly high ergonomic risks. Studies conducted in marble processing facilities consistently demonstrate sustained physical strain, improper lifting techniques, and frequent exposure to awkward postures such as bending, squatting, and reaching (9–11). Burdur Province in southwestern Türkiye is one of the country's major marble-producing regions, and the dense clustering of factories operating under similar high-risk ergonomic conditions makes it an important setting for evaluating interventions aimed at preventing LBP among marble workers (12).

The relationship between training and LBP outcomes is supported by both biomechanical and behavioral mechanisms. Improper lifting increases lumbar compression and shear forces, causes microtrauma to intervertebral discs, and contributes to inflammation, muscle fatigue, ligament laxity, and decreased spinal stability (13). Prospective studies have shown that lifting loads >25 kg or performing more than 25 lifts per day significantly increases both the incidence and severity of LBP (14), while workers lifting ≥30 kg face a substantially higher risk of acute occupational LBP compared with those lifting < 10 kg (15). Training programs focusing on posture correction, safe lifting techniques, and proper body mechanics help reduce trunk flexion angles, redistribute loads more efficiently, enhance neuromuscular coordination, and ultimately decrease mechanical loading on the lumbar spine. These changes reflect principles of motor learning, through which repeated, feedback-based practice leads to improved movement patterns and reduced biomechanical strain (13, 15–17). Therefore, national and international organizations recommend that workers be trained in protective and safe manual handling techniques, and publish guidelines, brochures, and posters to support this process (18–20).

In addition to biomechanical effects, training influences LBP through behavioral pathways. According to the Health Belief Model, individuals are more likely to adopt protective behaviors when they perceive themselves as susceptible to injury, recognize the severity of consequences, understand the benefits of safe practices, and develop sufficient self-efficacy through guided practice (21). Interactive, skills-based training—supported by demonstrations, corrective feedback, and supervised repetition—reinforces self-efficacy and facilitates the internalization of safer motor patterns. Printed educational materials serve as ongoing cues to action, further supporting retention of recommended behaviors (22–24). Evidence shows that occupational health nurse—led training enhances workers' awareness, behavioral adoption, and self-efficacy in preventing LBP (25–27).

Despite strong theoretical and empirical foundations, several important research gaps remain. First, ergonomic training studies focusing specifically on marble workers are scarce, despite their high exposure to hazardous manual handling. Second, few studies evaluate the full pathway from training to musculoskeletal outcomes by simultaneously assessing knowledge, observed manual handling behavior, and LBP severity. Third, there is limited sector- and region-specific evidence from settings such as Burdur, where occupational health nurses are rarely employed and educational materials related to back health are lacking. Fourth, the comparative effectiveness of different training modalities—such as interactive training vs. printed-only materials—has not been adequately examined under real industrial conditions, although this information is essential for designing scalable and context-appropriate interventions.

In this context, theory-informed, evidence-based training approaches may represent a practical strategy to reduce ergonomic risks in high-load industrial environments such as marble factories. Therefore, the present study aimed to compare the effects of two educational approaches—printed materials and interactive training supported by printed materials—on workers' knowledge of LBP prevention, manual handling behaviors, and LBP levels. By evaluating both behavioral and biomechanical pathways within a non-randomized experimental design, the study seeks to provide sector-specific evidence that may inform occupational health and safety practices, particularly in workplaces where occupational health nurses are not present. The hypotheses of the study are as follows:

1. The mean knowledge scores of workers in the intervention groups regarding low back health protection would increase more than those of the control group.
2. The mean manual handling behavior scores of workers in the intervention groups would increase more than those of the control group.
3. The mean VAS pain scores of workers in the intervention groups would decrease more than those of the control group.

## 2 Materials and methods

### 2.1 Study design

**Figure 1:**
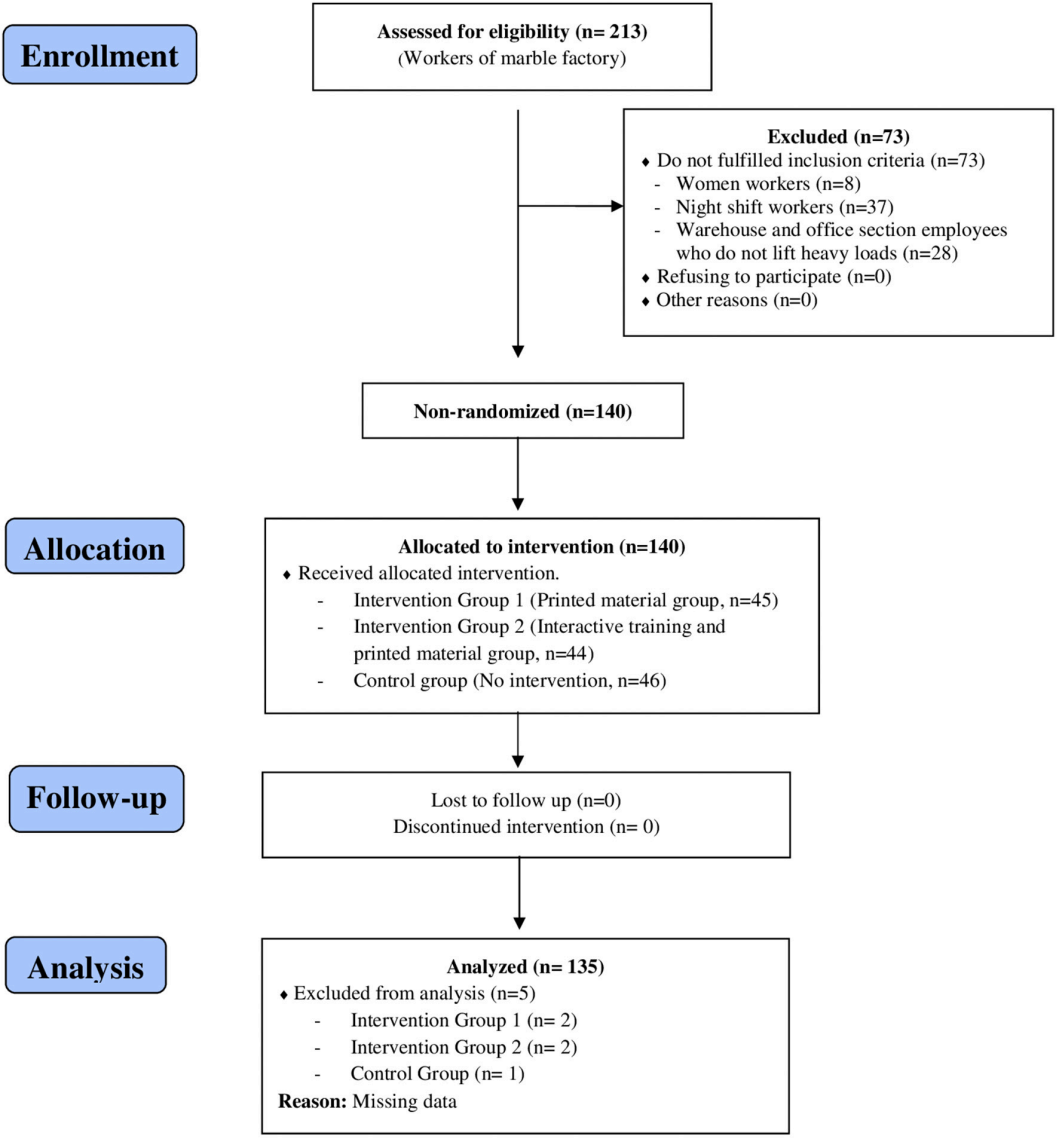
Trend flow diagram of the research.

This study was conducted as a non-randomized experimental (quasi-experimental) design with pretest, posttest, follow-up, and control groups, and was reported in accordance with the TREND guideline (Figure 1). The study was originally developed as part of a master's thesis.

### 2.2 Study setting and sample

The research was carried out in a marble factory located in the Bucak district of Burdur Province. The factory consisted of five independent production units (office, cutting, polishing, tile-processing, and warehouse/stock), each housed in separate buildings and operating under two shifts: 08:00–18:00 (day shift) and 18:00–03:30 (night shift). At the time of the study, the factory employed a total of 213 workers.

The minimum required sample size was calculated using G^*^Power 3.1.9.2 based on an effect size of 0.40 for comparisons among more than three groups, with a 95% confidence level, a Type I error of 0.05, and a Type II error of 0.10 (90% power). This calculation indicated a required sample of 84 participants. To allow for an estimated 20% non-response rate, the target sample size was increased to 102 participants (34 per group). Due to operational constraints and anticipated worker turnover during the COVID-19 period, a non-probability voluntary sampling approach was used. All workers who met the eligibility criteria and were present during the data collection period were invited to participate, and 135 workers consented and completed the study.

### 2.3 Study groups of research

The study included three production departments in which manual lifting and carrying of marble blocks were routinely performed. Each of these departments operated in separate physical areas with distinct work tasks and shift arrangements. To assign them to study groups, the three departments were randomly allocated to one of the groups by an independent researcher using a simple lottery procedure. The groups were as follows:

- **Intervention 1:** printed educational materials only (*n* = 45)
- **Intervention 2:** interactive training supported with printed materials (*n* = 44)
- **Control group:** no training during the study period (*n* = 46)

All eligible workers within each department were enrolled in the corresponding study group. The selected departments had comparable job characteristics and workload intensity to reduce baseline differences between groups.

### 2.4 Sample eligibility criteria

Workers were included in the study if they:

- were 18 years of age or older,
- had been employed in one of the heavy-lifting departments for at least three months,
- could read and write Turkish, and
- voluntarily agreed to participate.

All workers who met these criteria and were available during the data collection period were recruited into the study.

### 2.5 Data collection tools

Data were collected using a Descriptive Information Form, the Low Back Health Protection Information Form, and the Manual Handling Observation Form, all of which were developed by the researchers based on the relevant literature (23, 25–28). In addition, the Visual Analog Scale (VAS) was used to assess workers' low back pain levels.

Content validity of the newly developed instruments was evaluated using Lawshe's (30) method based on the assessments of eight experts. For each item, the Content Validity Ratio (CVR) was calculated, and for each instrument, the Content Validity Index (CVI) was computed. In line with the critical value recommended by Ayre and Scally (31), which specifies a minimum CVR of 0.75 for eight experts, all instruments met or exceeded the required threshold. The CVI values were 0.95 for the Low Back Health Knowledge Form, 1.00 for the Manual Handling Observation Form, 0.94 for the leaflet, and 0.95 for the poster (after revision of the title item), indicating satisfactory content validity.

Reliability testing was conducted for the Low Back Health Knowledge Form, which consists of 22 dichotomous (true–false) items. Item difficulty and discrimination indices were calculated using TAP software, and internal consistency was assessed using the Kuder–Richardson Formula 20 (KR-20). The mean item difficulty was 0.69, the mean discrimination index was 0.46, and the KR-20 coefficient was 0.80, demonstrating good internal consistency. These findings show that all instruments used in the study were valid and reliable.

#### 2.5.1 Descriptive information form

This form consists of 15 questions assessing participants' sociodemographic characteristics, health habits, working conditions, and history of low back pain.

#### 2.5.2 Low back health protection information form

This form was developed to assess participants' knowledge regarding low back health protection and consists of 22 statements (15 true and 7 false). Each correct answer is scored as “1,” while incorrect or “don't know” responses are scored as “0,” yielding a total score range of 0–22. Higher scores indicate greater knowledge of low back health protection. The form demonstrated good psychometric properties, with a mean item difficulty of 0.69, a discrimination index of 0.46, and a Kuder–Richardson (KR-20) reliability coefficient of 0.80.

#### 2.5.3 Manual handling observation form

This checklist contains 15 items evaluating the application steps of correct manual handling techniques. Each correctly performed step is scored as “1,” and each incorrect step is scored as “0,” resulting in a total score between 0 and 15. Higher scores reflect more accurate manual handling behavior. Inter-observer agreement was examined as part of the validity evidence. Three observers (the researcher, a workplace nurse, and a nurse academician) independently rated participant performance, and Fleiss' Kappa coefficients ranged from 0.99 to 1.00, indicating almost perfect agreement.

Prior to data collection, the observation conditions were standardized to ensure consistent assessment. A 10-kg marble block was used as the test load, and its lifting, carrying, and placement route was marked on the factory floor. Participants were asked to lift, carry, and replace the block along this path while being observed. The load mass and carrying distance were selected according to international ergonomic safety standards to ensure worker safety and minimize fatigue or injury risk.

#### 2.5.4 Visual analog scale (VAS)

The Visual Analog Scale (VAS) was used to measure the severity of low back pain. The scale consists of a 10-cm horizontal line anchored by “0 = no pain” and “10 = unbearable pain.” Participants were asked to mark the point that best represented their pain intensity. Scores were classified as follows: 0 = no pain; 1–2 = mild, annoying pain; 3–4 = irritating, distressing pain; 5–6 = unpleasant, awful pain; 7–9 = intense, severe pain; and 10 = unbearable pain. Higher scores indicate greater pain severity (29).

### 2.6 Pilot study

A pilot study was conducted with nine marble factory workers from a facility not included in the main sample between January 6 and 17, 2022, to evaluate the clarity, feasibility, and applicability of the data collection tools and the training procedures. The pilot confirmed that all instruments were understandable and could be completed without difficulty, and that the training and observation protocols were feasible under real factory conditions. Inter-observer agreement for the Manual Handling Observation Form was also examined, and items with lower agreement were identified. Based on these findings, a third observer was included and additional observer training was conducted prior to the main study. No further modifications to the instruments or procedures were required. The pilot study demonstrated that the overall research protocol was suitable for implementation in the main study.

### 2.7 Data collection

Data were collected at three time points between February and April 2022: before the intervention (pre-test), 1 week after the intervention (post-test), and 1 month after the post-test (follow-up). Questionnaires were administered to participants using a self-report format. On the same day as the questionnaire administration, the Manual Handling Observation Form was completed independently by three observers in accordance with the standardized observation protocol.

### 2.8 Intervention program

The intervention utilized three educational tools developed by the researchers: a PowerPoint presentation, a brochure, and a poster illustrating proper manual handling techniques. Expert opinions from eight specialists—including a physical therapist, physiotherapist, orthopedist, occupational health and safety specialist, occupational nurse, and a public health nursing academic—were obtained during the material development phase. All educational materials demonstrated acceptable content validity (CVI values: brochure = 0.94, poster = 0.95, presentation = 0.96; CVR critical = 0.75), confirming conceptual clarity and suitability for workplace ergonomic training (30, 31).

Following the pre-test, the interactive training group (Intervention 2) received two in-person training sessions in the factory meeting room within the same week. Session 1 included information on lumbar anatomy, causes of low back pain, and occupational risk factors. Session 2 focused on body mechanics, correct posture, and safe manual handling techniques. The “Tell–Show–Do” method was employed: the topic was first explained, demonstrated on a model, and then practiced by workers under supervision (as shown in the Supplementary material). At the end of the training, the brochure was distributed and the poster was displayed in all relevant work areas.

On the same day, the printed materials group (Intervention 1) was provided only with the brochure and poster, which contained the same content used in the interactive training.

The control group received no training or materials during the study period and was not informed about future training opportunities to avoid expectancy effects. After all data collection procedures were completed, the same educational materials were provided to the control group to ensure ethical compliance.

### 2.9 Preparation of the researcher and observers

The researcher completed accredited training in “Manual Handling and Carrying Techniques” and attended graduate-level courses in “Curriculum Development and Instructional Design” and “Mechanical Fundamentals of Movement” to support the development of the intervention content. Prior to data collection, the researcher provided training to the three observers on the use of the Manual Handling Observation Form, including a review of all items, scoring criteria, and a sample application to ensure consistency in the observation process.

### 2.10 Data evaluation

Data were analyzed using the Statistical Package for Social Sciences (SPSS, version 23). Normality was evaluated using outlier analysis and distributional checks. The homogeneity of the study groups at baseline was assessed using the Chi-square test. To examine the effectiveness of the interventions, between-group comparisons were performed using one-way analysis of variance (ANOVA), and within-group changes across the three measurement points were evaluated using repeated-measures ANOVA. When significant effects were detected, effect sizes were calculated using eta squared (η^2^) and interpreted as small (0.01), medium (0.06), and large (0.14) (University of Cambridge, 2021). All statistical analyses were two-tailed, and a *p*-value of < 0.05 was considered statistically significant.

## 3 Results

Of the participants, 33.3% were between 40 and 49 years of age, 75.6% were married, 37.1% had completed elementary school, and 45.2% reported low economic income (Table 1). Furthermore, 38.5% of participants were overweight, 22.2% had chronic illnesses, 46.7% were smokers, 57.8% exercised regularly, and 65.9% slept < 8 h per night. Additionally, 39.6% reported having worked for 1–5 years, and 65.9% reported having rest periods during working hours (Table 2). According to the groups of the study, no statistically significant difference was found in terms of the characteristics of the participants other than the marital status variable (*p* > 0.05) (Tables 1, 2). In addition, the pre-test low back health protection knowledge scores, manual handling observation scores and VAS scores of the participants did not differ statistically significantly according to the groups (*p* > 0.05) (Table 3).

**Table 1:** Comparison of sociodemographic characteristics of workers by groups.

**Variable**	**Level**	**Intervention group 1**		**Intervention group 2**		**Control group**		**Total**		**Test^#^** ***p*-value**
		***n***	**%**	***n***	**%**	***n***	**%**	***n***	**%**	
Age	19–29	12	26.7	8	18.2	11	23.9	31	22.96	0.695
	30–39	8	17.8	14	31.8	9	19.6	31	22.96	
	40–49	17	37.8	13	29.5	15	32.6	45	33.33	
	≥50 years	8	17.8	9	20.5	11	23.9	28	20.74	
Marital status	Married	30	66.7	40	90.9	32	69.6	102	75.56	**0.015** ^*^
	Single	15	33.3	4	9.1	14	30.4	33	24.44	
Education attainment	Primary school	15	33.3	19	43.2	16	34.8	50	37.04	0.697
	Middle school	18	40	13	29.5	14	30.4	45	33.33	
	High school	12	26.7	12	27.3	16	34.8	40	29.63	
Working year	1–5 years	9	20	14	31.8	17	37	40	29.63	0.396
	6–10 years	13	28.9	10	22.7	13	28.3	36	26.67	
	11–15 years	10	22.2	6	13.6	5	10.9	21	15.56	
	16 years	13	28.9	14	31.8	11	23.9	38	28.15	
Economical status	Does not meet basic needs	18	40	24	54.5	19	41.3	61	45.19	0.512
	Meets some basic needs	7	15.6	9	20.5	9	19.6	25	18.52	
	Above basic needs	20	44.4	11	25	18	39.1	49	36.3	

Intervention group 1= Printed material group, Intervention group 2= Interactive Education and printed material group, ^#^Test= Chi-square test, ^*^p < 0.05.

**Table 2:** Comparison of some descriptive characteristics of workers according to groups.

**Variable**	**Level**	**Intervention group 1**		**Intervention group 2**		**Control group**		**Total**		**Test^#^ *p*-value**
		***n***	**%**	***n***	**%**	***n***	**%**	***n***	**%**	
Rest time at work	Available	31	68.9	29	65.9	29	63	89	65.93	0.841
	Unavailable	14	31.1	15	34.1	17	37	46	34.07	
Working another job	Available	6	13.3	10	22.7	7	15.2	23	17.04	0.460
	Unavailable	39	86.7	34	77.3	39	84.8	112	82.96	
Diagnosis of low back pain	Available	10	33.33	7	21.88	8	28.57	25	27.78	0.599
	Unavailable	20	66.67	25	78.13	20	71.43	65	72.22	
Physical activity	Immobile	18	40	8	18.2	7	15.2	33	24.44	0.081
	Medium active	7	15.6	25	56.8	25	54.3	57	42.22	
	Active	20	44.4	11	25	14	30.4	45	33.33	
Body mass index	Normal weight	16	35.6	14	31.8	19	42.22	49	36.57	0.363
	Overweight	21	46.7	15	34.1	16	35.56	52	38.81	
	Obese	8	17.8	15	34.1	10	22.22	33	24.63	
Diagnosis of chronic disease	Available	9	20	11	25	10	21.7	30	22.22	0.847
	Unavailable	36	80	33	75	36	78.3	105	77.78	
Smoking	Available	16	35.6	23	52.3	24	52.2	63	46.67	0.187
	Unavailable	29	64.4	21	47.7	22	47.8	72	53.33	
Doing sport	Available	29	64.4	25	56.8	24	52.2	78	57.78	0.490
	Unavailable	16	35.6	19	43.2	22	47.8	57	42.22	
Sleeping time	< 8 h	26	57.8	28	63.6	35	76.1	89	65.93	0.170
	≥8 h	19	42.2	16	36.4	11	23.9	46	34.07	

Intervention group 1 = Printed material group, Intervention group 2 = Interactive Education and printed material group, ^#^Test = Chi-square test.

**Table 3:** Comparison of back health protection knowledge, manual handling observation and VAS scores of study groups.

**Variables**	**Test (time) #**	**Intervention group 1** ^**(1)**^		**Intervention group 2** ^**(2)**^		**Control group** ^**(3)**^		**Test (group)** ^**μ**^			**Test (group × time)^β^**
		**(Mean** ±**SD)**	**95% CI [LL, UL]**	**(Mean** ±**SD)**	**95% CI [LL, UL]**	**(Mean** ±**SD)**	**95% CI [LL, UL]**	***p*** **-value**	***Post-hoc***	η^2^	***p*** **-value**
Low back health protection knowledge scores	Pre-test^a^	13.7 ± 3.4	[12.7, 14.7]	14.0 ± 4.3	[12.7, 15.2]	12.3 ± 5.6	[10.6, 13.9]	0.167	–	–	**< 0.001** ^***^
	Post-test^b^	15.4 ± 2.7	[14.6, 16.2]	20.4 ± 0.7	[20.2, 20.6]	12.2 ± 5.6	[10.6, 13.9]	**0.009** ^*^	2 > 3	0.07	
	Follow-up^c^	15.0 ± 2.7	[14.2, 15.8]	19.7 ± 1.0	[19.3, 20.0]	12.6 ± 5.1	[11.0, 14.0]	**< 0.001** ^***^	2 > 1 > 3	0.43	
	Test (time)^#^										
	*p*-value	**< 0.001** ^***^		**< 0.001** ^***^		**0.013** ^*^					
	*Post-hoc*	b > c > a		b > c > a		c > b					
	η^2^	0.52		0.66		0.12					
Manual handling observation scores	Pre-test^a^	6.2 ± 2.3	[5.5, 6.9]	5.9 ± 1.8	[5.3, 6.4]	6.1 ± 1.9	[5.5, 6.6]	0.209			**< 0.001** ^***^
	Post-test^b^	7.2 ± 2.1	[6.6, 7.9]	13.9 ± 0.8	[13.7, 14.2]	6.2 ± 1.9	[5.7, 6.8]	**< 0.001** ^***^	2 > 1 > 3	0.80	
	Follow-up^c^	5.6 ± 1.9	[5.5, 6.1]	12.4 ± 1.2	[12.0, 12.8]	5.8 ± 1.8	[5.2, 6.3]	**< 0.001** ^***^	2 > 1, 3	0.77	
	Test (time)^#^										
	*p*-value	**0.002** ^**^		**< 0.001** ^***^		0.084					
	*Post-hoc*	b>c		b>c>a		-					
	η^2^	0.18		0.92		-					
VAS	Pre-test^a^	4.0 ± 3.2	[3.0, 5.0]	4.0 ± 3.0	[3.1, 4.9]	3.5 ± 2.8	[2.6, 4.3]	0.708	–		**< 0.001** ^***^
	Post-test^b^	3.5 ± 3.1	[2.6, 4.5]	2.0 ± 1.8	[1.4, 2.5]	3.0 ± 2.4	[2.3, 3.8]	**0.020** ^*^	1 > 2	0.06	
	Follow-up^c^	3.4 ± 3.1	[2.5, 4.4]	2.0 ± 1.8	[1.4, 2.5]	3.1 ± 2.5	[2.3, 3.8]	**0.024** ^*^	1 > 2	0.05	
	Test (time)^#^										
	*p*-value	**0.001** ^**^		**< 0.001** ^***^		**0.010** ^*^					
	*Post-hoc*	a > b, c		a > b, c		a > b					
	η2	0.20		0.60		0.12					

(1) Printed material group; (2) Interactive education and printed material group; (3) Control group. Superscript letters a, b, and c indicate statistically significant differences between time-points based on *post-hoc* comparisons, Test (time) #, Analysis of Variance in Repeated Measurements; Test (group) μ, One-way Analysis of Variance; Test (Group X Time) β, One-way Analysis of Variance; CI, Confidence Interval; LL, Lower Limit; UL, Upper Limit; η^2^, Eta-squared; ^*^p < 0.05, ^**^p < 0.01, ^***^p < 0.001.

### 3.1 Comparison of post-intervention low back health protection knowledge scores of the study groups

**Figure 2:**
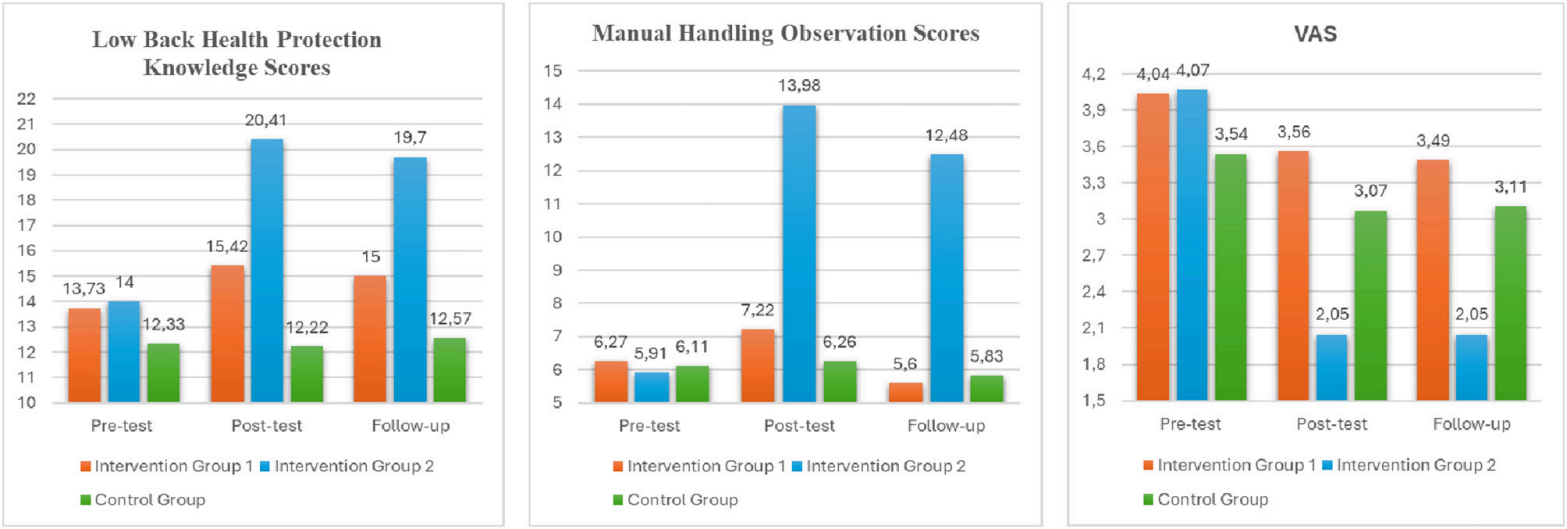
Comparison of pre-test, post-test, and follow-up test scores of the groups.

According to the ANOVA results, the participants mean scores for low back health protection knowledge differed significantly between groups, with medium to high effect sizes, both in the post-test (*F* = 4.93, *p* = 0.009, η^2^ = 0.07) and the follow-up test (*F* = 50.54, *p* < 0.001, η^2^ = 0.43). In *post hoc* analyses, the mean knowledge scores of the interactive education and printed material group (intervention 2) were significantly higher than the mean knowledge scores of the control group in the post-test and significantly higher than both the mean knowledge scores of the control group and the mean knowledge scores of the printed material group (intervention 1) in the follow-up test. The mean knowledge scores of the printed material group were also higher than those of the control group in the follow-up tests (Table 3, Figure 2).

When the time-dependent differentiation of the knowledge levels of the groups was examined, a significant difference was determined with a high effect size in both intervention groups (*F*_intervention1_ = 47.448, *p* < 0.001, η^2^ = 0.52; *F*_intervention2_ = 83.321, *p* < 0.001, η^2^ = 0.66). According to the *post hoc* tests, post-test and follow-up test scores of the intervention groups showed a significant increase compared to the pre-test scores, while a significant decrease was observed in the follow-up tests compared to the post-test (Table 3, Figure 2). There was also a statistically significant positive (increase) difference between the post-test and follow-up tests of the control group (*F* = 6.026, *p* = 0.013, η^2^ = 0.12).

When the comparison results related to the common effect of group and time were analyzed, it was found that the mean scores of the knowledge of protecting low back health differed statistically significantly (*F* = 60.947, *p* < 0.001) (Table 3).

### 3.2 Comparison of post-intervention manual handling observation scores of the study groups

The mean manual handling observation scores of the participants showed a significant difference at high effect level at post-test (*F* = 264.27, *p* < 0.001, η^2^ = 0.80) and follow-up test (*F* = 227.21, *p* < 0.001, η^2^ = 0.77). According to the *post hoc* analysis results, it was seen that the observation scores of the interactive training and printed material group (intervention 2) were significantly higher than the scores of the other groups both in the post-test and in the follow-up test. The manual handling observation scores of the printed material group (intervention 1) were also significantly higher than the control group in the follow-up test. In the time-dependent comparison results of the observation scores of the groups, a more than two-fold increase was observed in the mean observation score of the intervention 2 group after the training (*F* = 501.789, *p* < 0.001, η^2^ = 0.92) and the effect size of this difference was found to be quite high.

In the intervention 1 group, in which only printed material was used, although the increase in the post-test was lower, a statistically significant difference was found according to time (*F* = 9.650, *p* = 0.002, η^2^ = 0.18). According to the *post hoc* results, there was a negative (decrease) difference between the post-test and follow-up tests of both intervention groups. Despite this, the mean follow-up test scores of the intervention 2 group were significantly higher than the pre-test scores (Figure 2). A significant time-dependent difference was not observed in the scores of the control group (*p* > 0.05).

In the comparison based on the joint effect of group and time, it was determined that the mean scores of manual handling observation (*F* = 145.865; *p* < 0.001) increased statistically significantly (Table 3).

### 3.3 Comparison of post-intervention VAS scores of the study groups

The mean VAS scores of the participants were also significantly different between the study groups at both post-test (*F* = 4.20, *p* = 0.020, η^2^ = 0.06) and follow-up test (*F* = 3.82, *p* = 0.024, η^2^ = 0.05) with a medium effect. The decrease in the level of low back pain after the interventions in the interactive training and printed material group (intervention 2) was highly significant and considerably lower than the mean VAS score of the other two groups. In *post hoc* tests, the difference between the post-test and follow-up tests means VAS scores of the intervention 2 group and the printed material group (intervention 1) were statistically significant.

When the time-dependent comparison results of the VAS scores of the study groups were analyzed, statistically significant differences were found both in the intervention groups (*F*_intervention1_ = 11.252; *p* < 0.001; η^2^ = 0.20), (*F*_intervention2_ = 65.063; *p* < 0.001; η^2^ = 0.60) and in the control group (*F* = 6.174; *p* = 0.010; η^2^ = 0.12). According to the partial eta squared value calculated to look at the effect size of the significant difference, it was seen that the intervention groups had a high effect size, and the control group had a low effect size. When the paired *post hoc* comparison results were analyzed, it was found that there was a significant difference between the pre-test and post-test, and pre-test and follow-up tests of the intervention groups, and a negative (decrease) significant difference between the pre-test and post-test of the control group (Table 3, Figure 2).

According to the ANOVA result examining the joint effect of group and time, it was seen that the mean VAS scores (*F* = 19.440, *p* < 0.001) differed statistically significantly.

## 4 Discussion

Low back pain (LBP) remains one of the most prevalent and disabling musculoskeletal disorders worldwide, particularly among workers frequently engaged in repetitive lifting and manual handling tasks, such as those in the marble industry. This study demonstrated that both interactive and printed educational interventions were effective in improving workers' knowledge and behaviors related to low back health protection and in reducing low back pain. These findings support the study hypotheses and are consistent with previous research showing the positive impact of workplace-based training programs on musculoskeletal health. These findings align with previous workplace-based interventions. Comparatively, studies from Malaysia, Iran, Brazil, India, and Egypt have reported similar benefits, showing that culturally adapted and low-cost educational interventions can significantly improve manual handling practices and reduce LBP incidence (16, 32–34). These parallels reinforce the external validity of the present findings.

These observed changes can be interpreted within the framework of the Health Belief Model (HBM), one of the most widely applied theories in health behavior research. According to HBM, individuals' health-related actions are shaped by their perceptions of susceptibility, severity, benefits, and barriers, as well as cues to action and self-efficacy (35, 36). In this study, the educational interventions may have increased workers' perceived susceptibility and severity by emphasizing the personal and occupational risks associated with improper lifting techniques. Similarly, the perceived benefits of adopting correct posture and safe manual handling behaviors were reinforced through participatory learning and visually engaging printed materials. Moreover, these interventions may have enhanced workers' self-efficacy, helping them feel more confident and capable of protecting their back health during daily work activities. The high effect size (η^2^) observed in this study may reflect both this significant effect of the interventions and within-group similarity.

The reduction in pain intensity among marble workers after the intervention—though a secondary outcome—highlights the potential clinical and economic implications of preventive education. Prior research has shown that improved ergonomic behavior not only reduces pain but also lowers absenteeism and healthcare utilization (26, 37–39). In this study, pain intensity was assessed individually using the 10-point VAS, a validated instrument for occupational populations. However, VAS, being a subjective measure, may be influenced by individual pain perception, representing a limitation of the study. The reduction in pain levels observed among workers is a secondary but clinically important finding and likely results from increased knowledge, correct manual handling behaviors, and improved self-efficacy, which are core constructs of the HBM. Future studies could supplement self-report data with objective biomechanical measurements such as electromyography or motion analysis to improve the accuracy of pain assessment.

One of the unexpected findings of this study was the significant increase in knowledge scores and decrease in pain intensity observed in the control group. This change may be attributed to a mindfulness effect triggered by the data collection process itself. Completing the information form and being observed during manual handling tasks may have increased participants' awareness of their posture, lifting behavior, and back health, resulting in unintentional learning and behavioral self-correction. Such responses are consistent with the testing effect and the Hawthorne effect, where repeated assessments or the awareness of being observed can alter participants' cognition and behavior (38, 40, 41). Previous quasi-experimental workplace studies have similarly reported that observation and measurement procedures alone can yield modest but statistically significant improvements within control groups (42, 43). Therefore, the improvements observed in the control group in this study are interpreted as measurement-induced mindfulness and increased situational awareness rather than the result of a direct intervention effect. From a behavioral theory perspective, sustaining change requires continuous cues and reinforcement. In this study, although both intervention groups achieved significant post-test gains, a partial decline occurred during follow-up. This pattern mirrors previous findings indicating that in the absence of reinforcement, perceived susceptibility and motivation may wane over time (42, 43). Therefore, refresher sessions, visual cues such as posters, and managerial feedback can serve as ongoing stimuli for maintaining ergonomic awareness (22, 28).

Previous studies have shown that workplace interventions for LBP prevention employ various methods, including lectures, printed materials, video- or web-based education, and multimodal strategies (39, 44–46). Interactive education supported by printed materials—such as in this study—demonstrates the strongest and most sustained behavioral effects because it engages both cognitive and motor learning pathways (36, 47, 48). Although the effect of printed-only education is smaller, it still plays a valuable role, particularly in settings where face-to-face training is not feasible (49). In Turkey, small and medium-sized enterprises employing fewer than 50 workers are not legally required to appoint occupational health personnel (20). Therefore, reproducing and distributing validated printed materials through professional associations and trade unions could serve as a cost-effective strategy for scaling up preventive education. Moreover, integrating behavioral-science-based modules into occupational health nursing curricula and workplace safety policies could institutionalize preventive ergonomics education (35, 50).

From a policy and practice perspective, integrating theory-based educational approaches into occupational health and safety frameworks will help institutionalize behavior change education as a component of preventive healthcare. Health professionals—particularly occupational nurses—have a key role in implementing behavioral theory–based interventions to promote sustainable health behavior change in industrial settings. The findings emphasize the importance of active involvement of occupational health professionals and the integration of behavioral education at the policy level. Future research should examine the long-term effectiveness of these strategies, including digital and cross-sectoral applications, to strengthen preventive occupational health in developing countries such as Türkiye.

### 4.1 Strength and limitations

This study has several key strengths. Factory departments were randomly assigned to minimize selection bias, and the data collection tools and training materials underwent validity and reliability analyses to ensure measurement precision. Participants' manual handling behaviors were assessed under standardized conditions by three independent observers, increasing the reliability and objectivity of the behavioral assessments. Furthermore, the intervention was implemented in a real industrial setting, increasing its ecological validity and practical applicability to occupational health settings.

However, some limitations must be acknowledged. Because participants were recruited from a single factory, the generalizability of the findings is limited. Furthermore, individual-level randomization was not possible due to the high risk of contamination among workers in the same departments. Finally, low back pain severity was assessed using self-reported measures, which may be subject to response bias. Future studies could expand the sampling frame, include objective biomechanical indicators, and cover multiple workplaces to increase the robustness and external validity of the findings.

## 5 Conclusion

The results of this study highlight the effectiveness of different training methods in protecting low back health among workers engaged in heavy lifting and repetitive movements. The study demonstrated positive improvements in participants' knowledge of back health, manual handling behavior, and a reduction in low back pain levels. Based on these findings, it is recommended to provide face-to-face and interactive training, along with monitoring and consultancy services from occupational health nurses and other health professionals, to prevent work-related back pain. Where such interventions are not feasible, printed materials can be used as an alternative. Additionally, it should be remembered that taking appropriate technical and managerial measures to reduce heavy lifting remains the most effective strategy for preventing back pain.

## Data Availability

The raw data supporting the conclusions of this article will be made available by the authors, without undue reservation.
