# Supplementary material for: Effect of training on knowledge, behaviors, and low back pain among marble workers: non-randomized experimental study
Source: Front Public Health. 2026 Jan 23;13:1725015. doi: 10.3389/fpubh.2025.1725015 (PMC12876133; doi:10.3389/fpubh.2025.1725015)
Supplement: Supplementary file 1 [file Supplementary_file_1.docx]

| **Supplementary Table 1.** Content, duration, learning objectives, and activities of interactive training sessions | | | | | |
| --- | --- | --- | --- | --- | --- |
| **Training Session** | **Duration** | **Topics** | **Learning Objectives** | **Activities and Techniques** | **Materials** |
| Session 1 | 30 min | 1. Normal anatomical structure of the lumbar region 2. Causes and risk factors of low back pain:  a) Characteristics of the load  b) Physical strength requirements  c) Characteristics of the working environment  d) Job requirements  e) Individual risk factors | Workers will be able to: - Explain the normal anatomy of the lumbar region - Explain the causes and risk factors of low back pain - Identify risk factors related to the load, work, and individual characteristics | - Introduction to training content - Lecture and Q&A session - Group discussion and brainstorming - Distribution of written materials | Computer, Projector, Hand Brochure, Poster, Visual intelligence test (warm-up activity) |
| Session 2 | 45 min | Protection of back health: a) Proper posture b) Body mechanics c) Appropriate manual handling techniques | Workers will be able to: - Define body mechanics and proper posture - Demonstrate correct manual handling techniques - List recommendations for protecting low back health | - Explanation of the topic - Demonstration of correct posture using a model - Practice of manual handling techniques by workers - Feedback and summarization | Computer, Projector, Model, Box (Load) |
